# C-955-03. Effectiveness of a Discharge Checklist with Burn Patients

**DOI:** 10.1093/jbcr/irag033.152

**Published:** 2026-04-06

**Authors:** Catherine Freeman, Victoria Allen, Michael White, Carl Schneider

**Affiliations:** University of Cincinnati Medical Center, Cincinnati, OH; University of Cincinnati Medical Center, Cincinnati, OH; University of Cincinnati Medical Center, Cincinnati, OH; University of Cincinnati Medical Center, Cincinnati, OH

## Abstract

**Introduction:**

The purpose of this study was to determine if implementation of a discharge checklist was an effective tool in ensuring that education was provided to patients discharging home. Developed by rehabilitation and nursing staff, the checklist outlined skills that the patient or caregiver should be able to complete prior to discharge. The nursing skills included dressing changes, medication management, signs and symptoms of infection. Meanwhile, the therapy skills involved completing exercise programs, use of garments and splints, and monitoring for skin breakdown. The discharge checklist was posted in every patient’s room. The nurse and therapists would teach the relevant skills prior to discharge, marking the checklist once competence was achieved.

**Methods:**

A mixed methodology approach was utilized to assess the sustainability, effectiveness, and the cost of creating and utilizing the discharge checklist. Sustainability was assessed by auditing the checklist to determine whether it was completed prior to discharge. The effectiveness of the discharge checklist was evaluated through surveys administered during follow-up calls conducted 24 to 72 hours post-discharge. The survey included both quantitative methods and qualitative approaches. Meanwhile, cost analysis considered both staff time and materials.

**Results:**

A total of 30 patients were enrolled in this quality improvement study. Therapy staff demonstrated higher completion rates of the checklist compared to nursing staff; however, both disciplines achieved completion rates exceeding 50%. The checklist was less frequently completed for patients with smaller burn injuries and shorter hospital stays. Over 80% of burn survivors surveyed strongly agreed that that the checklist was helpful in ensuring a safe discharge home. Whereas 64% of the survivors strongly agreed that they were confident with their dressing change on discharge. 76% of the survivors felt that the education that they received on the burn rehabilitation program was very good. The follow-up phone call required less than five minutes of staff time, while the production cost of each checklist was approximately $2 to $3.

**Conclusions:**

Implementation of a checklist was a cost-effective way to standardized discharge education provided to patients returning home. Staff were able to utilize the checklist to ensure that patients were receiving education on their dressings, medication management, positioning, splinting, exercise program, and outlets for psychosocial support. By ensuring patients received this education we were able to promote continuity of care and support a safe transition to home.

**Applicability of Research to Practice:**

With survival rates increasing a comprehensive approach is required to ensure patient success with their burn recovery. A discharge checklist sets clear guidelines to help ensure patients achieve all the necessary education to be successful at home.

**Funding for the study:**

N/A.

